# Ventricular Tachycardia Has Mainly Non-Ischaemic Substrates in Patients with Autoimmune Rheumatic Diseases and a Preserved Ejection Fraction

**DOI:** 10.3390/diagnostics11030519

**Published:** 2021-03-15

**Authors:** George Markousis-Mavrogenis, George Poulos, Theodoros Dimitroulas, Aikaterini Giannakopoulou, Clio Mavragani, Vasiliki Vartela, Dionysia Manolopoulou, Genovefa Kolovou, Paraskevi Voulgari, Petros P. Sfikakis, George D. Kitas, Sophie I. Mavrogeni

**Affiliations:** 1 Onassis Cardiac Surgery Center, 17674 Athens, Greece; georgemm32@gmail.com (G.M.-M.); gdpoulos@yahoo.com (G.P.); vasvartela@yahoo.gr (V.V.); dmanolopoulou@yahoo.com (D.M.); genovefa@kolovou.com (G.K.); 2 Department of Internal Medicine, Rheumatology, Aristotle University, 54124 Thessaloniki, Greece; dimitroul@hotmail.com; 3 Department of Paediatric Cardiology, Agia Sofia Children Hospital, 11527 Athens, Greece; aikaterinigiannakopoulou@hotmail.com; 4 Pathophysiology Department, Laikon Hospital, 11527 Athens, Greece; kmauragan@med.uoa.gr; 5 First Department of Propaedeutic Internal Medicine and Joint Rheumatology Program, Medical School, National and Kapodistrian University of Athens, 15772 Athens, Greece; psfikakis@med.uoa.gr; 6 Rheumatology Clinic, University of Ioannina, 45110 Ioannina, Greece; pvoulgar@uoi.gr; 7 First Department of Propeudeutic and Internal Medicine, Laikon Hospital, 11527 Athens, Greece; 8 Dudley Group NHS Foundation Trust, Dudley DY1 2HQ, UK; gkitas@hygeia.gr; 9 School of Sport, Exercise and Rehabilitation Sciences, University of Birmingham, Birmingham B15 2TT, UK

**Keywords:** oedema, fibrosis, cardiovascular magnetic resonance, rhythm disturbance, myocarditis, ischaemia, sudden cardiac death

## Abstract

Non-sustained ventricular tachycardia (NSVT) is a potentially lethal arrhythmia that is most commonly attributed to coronary artery disease. We hypothesised that among patients with NSVT and preserved ejection fraction, cardiovascular magnetic resonance (CMR) would identify a different proportion of ischaemic/non-ischaemic arrhythmogenic substrates in those with and without autoimmune rheumatic diseases (ARDs). In total, 80 consecutive patients (40 with ARDs, 40 with non-ARD-related cardiac pathology) with NSVT in the past 15 days and preserved left ventricular ejection fraction were examined using a 1.5-T system. Evaluated parameters included biventricular volumes/ejection fractions, T2 signal ratio, early/late gadolinium enhancement (EGE/LGE), T1 and T2 mapping and extracellular volume fraction (ECV). Mean age did not differ across groups, but patients with ARDs were more often women (32 (80%) vs. 15 (38%), *p* < 0.001). Biventricular systolic function, T2 signal ratio and EGE and LGE extent did not differ significantly between groups. Patients with ARDs had significantly higher median native T1 mapping (1078.5 (1049.0–1149.0) vs. 1041.5 (1014.0–1079.5), *p* = 0.003), higher ECV (31.0 (29.0–32.0) vs. 28.0 (26.5–30.0), *p* = 0.003) and higher T2 mapping (57.5 (54.0–61.0) vs. 52.0 (48.0–55.5), *p* = 0.001). In patients with ARDs, the distribution of cardiac fibrosis followed a predominantly non-ischaemic pattern, with ischaemic patterns being more common in those without ARDs (*p* < 0.001). After accounting for age and cardiovascular comorbidities, most findings remained unaffected, while only tissue characterisation indices remained significant after additionally correcting for sex. Patients with ARDs had a predominantly non-ischaemic myocardial scar pattern and showed evidence of diffuse inflammatory/ischaemic changes (elevated native T1-/T2-mapping and ECV values) independent of confounding factors.

## 1. Introduction

In patients with autoimmune rheumatic diseases (ARDs), development of arrhythmogenic inflammatory cardiomyopathy can lead to out-of-hospital cardiac arrest (OHCA) and can also potentially cause sudden cardiac death (SCD) [1,2]. An important challenge in the context of OHCA is that the majority of patients experience it as a first presentation, often without previous symptoms or a diagnosed cardiac condition [3]. This makes primary prevention a daunting task. Different forms of ventricular tachycardia (VT) and fibrillation (VF) reportedly account for ~75% of OHCA cases [4]. In patients with ARDs, instances of VT/VF secondary to (occult) cardiac involvement are not uncommon. The occurrence of VT/VF is prevalent in numerous ARDs and has been described, among others, in systemic lupus erythematosus (SLE), rheumatoid arthritis (RA), polymyalgia rheumatica (PMR), giant cell myocarditis (GCM) and systemic sclerosis (SSc) [5,6,7,8,9]. This arrhythmogenicity was traditionally attributed to ventricular scarring leading to re-entrant VT/VF [10]. However, a complex interplay between the immune system and the heart may lead to arrhythmogenicity via other routes in patients with ARDs, namely microvascular coronary artery disease, myocardial oedema, immune cell infiltration and direct effects of cytokines and immunoglobulins on cardiac ion channels [1]. These factors may adversely affect the conductive properties of the myocardium, thus causing pathological alterations in excitability and subsequent arrhythmogenicity. Notably, OHCA in these cases may be the first presentation of the disease or may occur after diagnosis, even if the underlying ARD is quiescent or the left ventricular ejection fraction (LVEF) is preserved [10,11].

Cardiovascular magnetic resonance (CMR) is a non-invasive imaging modality that does not utilise ionising radiation and excels in the cardiac evaluation of patients both with and without ARDs. Specifically, CMR can characterise myocardial tissues with regard to the presence of oedema and fibrosis, as well as more aspecific expansion of the extracellular space [11], all of which may elucidate underlying ischaemic or non-ischaemic/inflammatory pathologic changes based on the observed pattern. As already stated, ischaemic heart disease is thought to pave the way for most cases of OHCA. However, the relative contribution of other arrhythmogenic substrates in relation to cardiac ischaemia, particularly in patients with ARDs, is not well described, even though non-ischaemic substrates may require different therapeutic approaches [12,13]. We hypothesised that in a mixed population of patients with a known occurrence of non-sustained VT (NSVT) and a preserved LVEF, CMR would identify a significantly different proportion of ischaemic and non-ischaemic arrhythmogenic substrates in patients with and without ARDs. Therefore, by using CMR, we examined potential arrhythmogenic substrates leading to NSVT in patients with and without ARDs and a preserved LVEF, while also aiming to identify CMR indices that can optimally discriminate between the two groups.

## 2. Significance and Innovation

In this manuscript, we present for the first time a comparison of patients with a recent history of non-sustained ventricular tachycardia and a preserved left ventricular ejection fraction with and without a diagnosis of ARDs. These were evaluated using CMR and X-ray coronary angiography when necessary. CMR evaluation demonstrated that patients with ARDs had a predominantly non-ischaemic myocardial scar pattern and showed evidence of diffuse inflammatory/ischaemic changes (elevated native T1/T2 mapping and ECV values), while patients without ARDs had myocardial fibrosis characteristic of macrovascular coronary artery disease. These findings were independent of confounding factors and may have significant implications for therapeutic approaches in patients with ARDs.

## 3. Patients and Methods

### 3.1. Patients

All participants with ARDs were prospectively recruited based on having experienced a recent episode of NSVT (last 15 days), as assessed either by a 24-h Holter recording or by electrocardiography upon presentation at their treating physician, and having a preserved LVEF (≥55%), based on bedside echocardiography. NSVT was defined as having three or more consecutive ventricular beats at a rate of >100 beats per minute with a duration < 30 s. In total, 40 consecutive patients with various ARDs were recruited. In addition, from a pool of patients that met the aforementioned criteria, underwent CMR and were diagnosed with non-ARD-related cardiac conditions, an additional 40 were 1:1 matched for age to the 40 patients with ARDs. The exact composition of each group is presented in Table 1. The median (interquartile range) values of QRS complexes associated with NSVT episodes were 8.5 (7, 10.5) and 8 (7, 10.5) for the non-ARD and ARD groups, respectively (*p* = 0.534). All participants reported palpitations at presentation and also presented with either angina pectoris or atypical chest discomfort (ARD group: 12.5% (5) vs. 87.5% (35), respectively; non-ARD group: 85% (34) vs. 15% (6), respectively). No participants reported a prior history of syncope or presented to their treating physician due to syncope. In addition, no participants experienced an OHCA, none had peripheral vascular disease or a previous coronary artery revascularisation and none had a cardiac pacemaker implanted.

All participants were examined with CMR within 15 days of the NSVT episode, in the context of evaluation for arrhythmogenic substrates. All patients with ARDs had a previously established diagnosis of ARD, while all non-ARD patients presented with NSVT without other known cardiac conditions. In the latter cases, CMR was used in combination with clinical evaluation to diagnose the underlying cause of the NSVT. The study was approved by the Onassis Cardiac Surgery Center’s medical ethics committee (415/23.11.09, 23 November 2009) and all participants provided written informed consent before study inclusion.

### 3.2. Methods

CMR examinations were performed using a 1.5-T scanner (Ingenia, Philips Medical Systems, Best, The Netherlands). The CMR protocol included standard steady-state free-precession cine CMR, black-blood T2-weighted short tau inversion recovery (STIR-T2) images, T1-weighted spin-echo early gadolinium enhancement (EGE) images and phase-sensitive inversion recovery late gadolinium enhancement (LGE) images, as described previously [10]. A dose of 0.1 mmol/kg gadobenate dimeglumine contrast medium was injected for EGE images and another 0.1 mmol/kg for late LGE images, according to the protocol suggested by the Lake Louise criteria [14].

T1 mapping was performed using a modified Look-Locker inversion recovery (MOLLI) sequence with a 3(3)5 scheme on 3 representative short-axis positions immediately before and 15 min after contrast medium administration. T2 mapping was performed on 3 corresponding LV short axes using a black-blood prepared, navigator-gated, free-breathing hybrid gradient (echo planar imaging) and spin-echo multiecho sequences [15].

### 3.3. CMR Data Analysis

The short-axis steady-state free-precession cine CMR was used to evaluate biventricular function (volumes and ejection fractions) according to the standard protocol [14]. Global myocardial inflammation was assessed on T2-weighted images by calculating the T2 signal intensity ratio as signal intensity of myocardium divided by signal intensity of skeletal muscle [14]. Global relative enhancement was calculated by measuring myocardial signal intensity on pre- and post-contrast T1-weighted spin-echo images relative to skeletal muscle [14]. The presence and pattern of LGE lesions were qualitatively assessed by consensus agreement of 2 experienced observers and expressed as a percentage of left ventricular mass (%LGE). Native and post-contrast T1 mapping, extracellular volume fraction (ECV), and T2 mapping were calculated as described previously [15], based on the mean value of 3 short-axis slices. For ECV, a cut-off of >29% was used for defining abnormal values [16].

### 3.4. X-Ray Coronary Angiography (XCA)

XCA was performed in all patients with identified subendocardial or transmural LGE, traditionally characterised as ischaemic patterns [17], to determine coronary artery patency.

### 3.5. Statistical Analysis

Statistical analyses were carried out with Stata SE v.16 (StataCorp. 2019. Stata Statistical Software: Release 16. StataCorp LLC., College Station, TX, USA). Normality of variables was determined using Q-Q plots and histograms when necessary. Normally distributed variables are presented as mean (standard deviation), not-normally distributed continuous variables are presented as median (interquartile range) and binary/categorical variables are presented as n (%). Differences in CMR variables between patients with and without ARDs were investigated with independent sample t-tests for normally distributed variables, Mann–Whitney *U* tests for continuous not-normally distributed variables and chi-square tests for categorical or binary variables. Logistic regression analysis was used to examine the effect of confounding factors (demographics and cardiovascular comorbidities). Statistical significance for all tests was considered for *p* ≤ 0.05.

## 4. Results

Baseline characteristics for the groups of patients with and without ARDs are presented in Table 1. The study population of 40 patients with and 40 without ARDs did not differ significantly in age (48.5 (12.5) vs. 47.8 (15.7) years, *p* = 0.83), but the group of ARD patients had a significantly higher proportion of females compared with those without ARDs (32 (80%) vs. 15 (38%), *p* < 0.001). All patients were scored as class I or II using the New York Heart Association (NYHA) functional classification. Median LVEF based on CMR was 63.0% (IQR: 60.5, 68.0) in patients with ARDs vs. 62.5% (56.5, 68.5) in patients without ARDs (*p* = 0.41). In the ARD group, the majority of patients had a known diagnosis of either SSc, SLE (*n* = 10 (25%) for both) or small vessel vasculitis (*n* = 6 (15%)), with less frequent cases of sarcoidosis, rheumatoid arthritis and ankylosing spondylitis (*n* = 4 (10%) for all). In the non-ARD group, the combination of CMR and clinical findings most frequently led to a diagnosis of infectious myocarditis in 12 (31%) patients, CAD in nine (23%) patients, takotsubo cardiomyopathy in four (10%) patients and hypertrophic or dilated cardiomyopathy in three (8%) patients each. Univariable comparisons of CMR findings between the two groups (Table 1) identified significantly higher left and right ventricular volumes in patients with ARDs compared to those without ARDs. However, LV and RV systolic function was comparable between the two groups. No changes in T2 signal ratio, EGE or LGE were identified between the two groups. In contrast, all parametric CMR indices differed significantly between the two groups. Namely, compared with patients with ARDs, those without ARDs had significantly lower median native T1 mapping (1078.5 (1049.0–1149.0) vs. 1041.5 (1014.0–1079.5), *p* = 0.003), significantly higher post-contrast T1 mapping (353.8 (59.5) vs. 427.6 (59.2), *p* < 0.001), significantly lower ECV (31.0 (29.0–32.0) vs. 28.0 (26.5–30.0), *p* = 0.003) and significantly lower T2 mapping (57.5 (54.0–61.0) vs. 52.0 (48.0–55.5), *p* = 0.001). Similarly, the proportion of patients with pathologic parametric CMR indices based on locally determined cut-off values was significantly higher in the ARD group, particularly for ECV and T2 mapping (Table 1).

The proportion of patients with identified LGE during their CMR examination trended towards lower values in the ARD group compared with the non-ARD group but did not reach statistical significance (21 (53%) vs. 29 (73%), *p* = 0.065). Nevertheless, in patients without ARDs, the pattern of LGE lesions was most commonly suggestive of an ischaemic lesion (transmural or subendocardial LGE following the distribution of coronary arteries), while in patients with ARDs, the LGE patterns were most commonly suggestive of a non-ischaemic distribution (Table 2). Namely, 13/21 (61.9%) patients with ARDs and identified LGE during CMR had a patchy inferolateral LGE pattern, 6/21 (28.6%) had a diffuse subendocardial fibrosis pattern and only 2/21 (9.5%) had a typical transmural (ischaemic) LGE pattern. Of the 29 patients without ARDs and identified LGE during CMR, 13 (44.8%) had transmural LGE, while the remainder had either subepicardial (12 patients (41.4%)) or diffuse subendocardial LGE (6 patients (20.7%)). The XCA revealed CAD in all participants of either group with transmural LGE. The two patients with ARDs and transmural LGE had significant left anterior descending (LAD) or right coronary artery (RCA) stenosis. Of the 13 patients without ARDs and transmural LGE, 10 had both significant LAD and RCA stenosis while three had significant left circumflex (LCx) coronary artery stenosis. However, no participants with diffuse subendocardial fibrosis presented evidence of CAD.

Univariable and multivariable logistic regression analyses for discriminating between the two groups were performed to investigate the effect of potential confounding and the results are presented in Table 3. All available CMR indices were examined in univariable analysis and subsequently corrected in a stepwise manner, first for age, hypertension, smoking (last 5 years), family history of CAD/CVD and hypercholesterolemia, and subsequently for the latter variables with the addition of sex. In the first step of multivariable corrections, biventricular volumes as well as native T1 mapping, post-contrast T1 mapping, ECV and T2 mapping remained significant predictors of group membership. However, when also corrected for sex, native T1 mapping and post-contrast T1 mapping were the only indices that significantly predicted group membership independent of the aforementioned confounding factors. When, instead of ECV as a continuous variable, we investigated the locally used cut-off value (>29%) for determining pathologic ECV values as a variable for determining group membership, this remained significant even after correction for all aforementioned confounding factors, including sex (Table 3). In addition, correcting for sex also led to RVEF reaching statistical significance, even though no differences were identified in univariable analyses. We additionally investigated whether native T1 mapping, post-contrast T1 mapping, ECV and T2 mapping showed incremental discriminatory value independent of LVEDV, LVESV and LVEF. Only native T1 mapping and post-contrast T1 mapping significantly discriminated between the two groups independent of the aforementioned indices (odds ratio (OR) (95% confidence interval) per 10-unit change: 1.09 (1.01–1.19), *p* = 0.030 and 0.82 (0.73–0.92), *p* = 0.001, respectively). T2 mapping had a non-significant trend towards group discrimination when correcting for these indices (1.08 (1.00–1.16), *p* = 0.054), while ECV did not significantly discriminate between the two groups when corrected for these indices (1.02 (0.92–1.11), *p* = 0.733).

A sensitivity analysis was performed for T1-based indices (native/post-contrast T1 mapping, ECV) by excluding patients with XCA-confirmed CAD; these were two (5%) patients from the ARD group and 13 (32.5%) patients from the non-ARD group. The analysis confirmed that native and post-contrast T1 mapping values remained significant univariable discriminators between the two groups even when patients with XCA-confirmed CAD were excluded (OR (95% CI) per 10-unit change: 1.11 (1.02–1.21), *p* = 0.012 and 0.79 (0.70–0.90), *p* < 0.001, respectively). These findings also remained significant after multivariable correction for age, hypertension, smoking, family history of CAD/CVD and hypercholesterolemia (1.12 (1.03–1.22), *p* = 0.010 and 0.79 (0.69–0.90), *p* < 0.001, respectively). However, ECV showed only a non-significant trend towards significant prediction of group membership in this sensitivity analysis, in both univariable (1.09 (0.97–1.22), *p* = 0.166) and multivariable (1.09 (0.96–1.22), *p* = 0.175) testing.

## 5. Discussion

In this study, we evaluated a cohort of 80 consecutive patients with NSVT and preserved LVEF, 40 with various previously diagnosed ARDs and 40 with non-ARD-related cardiac conditions. Biventricular systolic function was preserved and did not differ significantly between the two groups. We identified numerous differences in CMR indices between the groups in univariable analyses, including biventricular volumes, native T1 mapping, post-contrast T1 mapping, T2 mapping and ECV. Furthermore, we identified significant differences in the pattern of myocardial fibrosis between the two groups, with the predominant pattern being non-ischaemic in patients with ARDs. Lastly, we investigated the effect of confounding. When accounting for age and cardiovascular comorbidities, most findings remained unaffected. When additionally corrected for sex, only native T1 mapping, post-contrast T1 mapping and ECV >29% remained significant predictors of group membership; RVEF became a significant predictor only after correcting for sex. It should be noted, however, that a higher proportion of females among patients with ARDs is not an unexpected finding, which is why the correction for sex was performed as a second, independent step.

There is increasing scientific interest regarding the use of CMR in non-ARDs with concomitant VT [18,19]. However, studies with CMR in the context of arrhythmogenicity in ARDs are less common. To our knowledge, most of the available literature on this topic has been published by our group and supports the notion that both myocardial oedema and fibrosis, as assessed by T1/T2 mapping and ECV, offer additional utility beyond LVEF in the evaluation of patients with ARDs who are at risk for VT [10], while also having significant predictive value for future high-risk arrhythmic events [20]. The findings of this investigation add to this expanding evidence pool and have important implications for the therapeutic considerations in patients with ARDs. Namely, according to current practice guidelines, prophylactic implantable cardioverter defibrillator implantation is indicated in patients with VT and an LVEF < 35% [21]. However, there is no guarantee that potentially lethal cardiac rhythm disturbances will not manifest in the presence of preserved LVEF, as was the case in all recruited patients with ARDs in this study. Our study demonstrates that the arrhythmogenic substrate in patients with ARDs and a preserved LVEF is mainly due to diffuse inflammatory/ischaemic fibrotic lesions and not classical CAD, as exemplified by significantly higher native T1 mapping, T2 mapping and ECV values in the ARD group, independent of confounding factors. In addition, the most prevalent myocardial fibrosis patterns observed in the ARD group are characteristic of diffuse microvascular disease, as is commonly seen in small-vessel vasculitis, SSc and SLE with anti-phospholipid syndrome [22]. The finding that patients with ARDs and NSVT have, on average, higher T1 mapping and ECV values compared with non-ARD patients with NSVT is important in the clinical context. This is because it emphasises the need for stricter control of systemic inflammation using immunomodulatory treatment, as well as the need for timely introduction of cardioprotective medication that can ameliorate microvascular disease in the heart. The finding that ECV values have only a non-significant trend towards predicting group membership in a separate sensitivity analysis excluding patients with confirmed CAD is probably attributable to insufficient statistical power resulting from the exclusion of ~19% of the study population, as this was not the case for native and post-contrast T1 mapping.

Interestingly, patients with ARDs are traditionally thought to primarily have a high incidence of classical CAD and most therapeutic approaches are focused on modifying cardiovascular risk factors [23]. However, numerous studies with CMR in various ARDs have already reported that cardiac lesions in these patients are indeed primarily of non-ischaemic aetiology [24,25,26], a finding which is expanded to ARDs with concomitant VTs specifically, based on our results. Without undermining the role of traditional preventive approaches for classical CAD in patients with ARDs, the development of novel and more efficient diagnostic and therapeutic protocols for better identification and control of arrhythmogenesis in these patients needs to be emphasised. Furthermore, this study, taken together with existing evidence, suggests that any VT episode in patients with ARDs should motivate additional testing with CMR, particularly including LGE and novel tissue characterisation indices, in order to identify the corresponding arrhythmogenic substrate and take additional preventive measures [15]. Lastly, autonomic neuropathy should be considered as a potential cause for rhythm disturbances leading to SCD in such cases [27]. This disorder manifests as sweating disturbances, gastrointestinal abnormalities and bladder and/or erectile dysfunction [27]. However, no patients in our study experienced similar symptoms and therefore autonomic neuropathy was not considered as a potential diagnosis.

Although the significantly higher tissue characterisation indices in patients with ARDs in our study, as well as the predominantly non-ischaemic myocardial fibrosis pattern, give an indication as to the nature of the underlying arrhythmogenic substrate, the exact aetiology of arrhythmogenicity in ARDs is not very well understood. Current evidence robustly indicates that, in ischaemic heart disease (IHD), strands of surviving myocardium at the periphery of the infarct region promote arrhythmogenicity via re-entry and have a heterogeneous appearance on CMR [28,29,30]. The aetiology of VT in non-ischaemic heart disease (NIHD) is less well understood, primarily due to the underlying pathology resulting from a variety of pathophysiological processes rather than a more uniform cause as in the case of ischaemia. The combination of focal myocardial fibrosis creating regions of conduction block, with non-uniform anisotropy and slow conduction through interstitial fibrosis, may promote re-entry and result in sustained ventricular arrhythmias [31]. Furthermore, myocytes within areas of interstitial fibrosis may spontaneously depolarise during diastole, resulting in abnormal automaticity and arrhythmia [31]. More recently, a plethora of additional interweaving mechanisms, such as immune cell infiltration of the myocardium and pro-arrhythmogenic effects of cytokines and antibodies, have been recognised as additional facets of the underlying multifactorial pathophysiology of arrhythmogenicity in NIHD [1]. In these patients, there is often mid-wall or subepicardial fibrosis, making ablation of arrhythmogenic loci challenging and potentially explaining why outcomes of ablation for control of arrhythmogenicity in NIHD are worse compared with IHD [32].

Previous studies have already demonstrated the added utility of novel tissue characterisation indices. In both IHD and NIHD, myocardial T1 mapping incrementally improved risk stratification in a model including LVEF, QRS duration and LGE [33]. In a similar study using ECV, high ECV values were associated with mortality [34]. Furthermore, ECV is of particular interest in patients with NIHD but without identifiable LGE, since it is a surrogate marker of diffuse interstitial fibrosis [10], and may also have value in further characterising the density of discrete scars, although robust data are still limited. In addition, acute damage to the myocardium may lead to myocardial oedema, which can be identified using both native T1 and T2 mapping. T1 mapping was recently also proven to be a valuable index in the evaluation of patients with ARDs and various systemic diseases [10] as well as cardiac sarcoidosis in particular [35]. Regarding the role of RVEF, previous studies suggest that RVEF may be impaired in various ARDs [36,37]. However, data regarding the predictive capacity of RVEF for mortality in patients with ARDs are rather scarce. According to a CMR study in patients with sarcoidosis, RV systolic dysfunction but not RV LGE was independently associated with all-cause mortality [38]. Nevertheless, it should be kept in mind that there were no differences in biventricular function in our study population after univariable testing, and RVEF could only significantly discriminate between the two groups when correcting for sex. Thus, the reliability of this association should be taken into account when interpreting these findings. Lastly, the identified differences in biventricular volumes can probably be attributed to the larger proportion of female patients in the ARD group.

## 6. Limitations

This study had the following limitations:

1. NSVT was an inclusion criterion. Evaluation of de novo VT occurrence after CMR is required to provide more robust evidence in support of the findings observed in this study. Our group has already published similar work in patients with SSc [20].
2. Potential differences between groups in some CMR indices might have not been identified due to insufficient statistical power, as in the case of LGE. In addition, the study did not include any patients with psoriatic arthritis.
3. A referral bias may have occurred since only severely ill patients with a well-documented history of NSVT were referred to our tertiary centre.
4. This study was performed in a population made up of patients with different ARDs. Further studies in uniform populations with individual ARDs should be performed before final conclusions can be drawn. Again, our group has already published similar work in patients with SSc [20].
5. The evaluation of myocardial strain in non-contrast CMR scans was not a part of this investigation.
6. The prevalence of CAD-related LGE might have been underestimated due to the relatively young age of the examined patient group.

## 7. Conclusions

In a cohort of 80 consecutive patients with NSVT and preserved LVEF, 40 with various previously diagnosed ARDs and 40 with non-ARD-related cardiac conditions, characterisation of myocardial fibrosis with CMR identified a non-ischaemic and ischaemic LGE pattern as the most predominantly occurring fibrotic pattern in the former and latter, respectively. Patients with ARDs were found to have significantly elevated native/post-contrast T1 mapping and ECV, which persisted in statistical significance after adjustment for confounding factors.

## Figures and Tables

**Table 1:** Baseline characteristics compared between patients with NSVT and normal LVEF, with or without ARDs. * *p* ≤ 0.05.

Variables		Patients with ARDs	Patients without ARDs	*p*-Value
Number of patients		40	40	N/A
Demographics		32 (80%) 48.5 (12.5)	15 (38%) 47.8 (15.7)	<0.001 * 0.83
	Female Sex			
	Age (years)			
Known ARD diagnosis				<0.001 *
	Systemic Sclerosis	10 (25%)	0 (0%)	
	Systemic Lupus Erythematosus	10 (25%)	0 (0%)	
	Sarcoidosis	4 (10%)	0 (0%)	
	Rheumatoid Arthritis	4 (10%)	0 (0%)	
	eGPA	4 (10%)	0 (0%)	
	Ankylosing Spondylitis	4 (10%)	0 (0%)	
	GPA	2 (5%)	0 (0%)	
	Takayasu Arteritis	1 (3%)	0 (0%)	
	Adamantiades–Behcet Disease	1 (3%)	0 (0%)	
Non-ARD Diagnosis Based on CMR and Clinical Findings				<0.001 *
	Infectious Myocarditis	0 (0%)	12 (31%)	
	CAD	0 (0%)	9 (23%)	
	Takotsubo Cardiomyopathy	0 (0%)	4 (10%)	
	Dilated Cardiomyopathy	0 (0%)	3 (8%)	
	Hypertrophic Cardiomyopathy	0 (0%)	3 (8%)	
	ARVC	0 (0%)	2 (5%)	
	Myopericarditis	0 (0%)	2 (5%)	
	Non-Compaction Cardiomyopathy	0 (0%)	2 (5%)	
	NSVT of Unknown Aetiology	0 (0%)	1 (3%)	
	Aortic Stenosis	0 (0%)	1 (3%)	
	Mitral Regurgitation	0 (0%)	1 (3%)	
CMR Indices				
	LVEDV (mL)	121.0 (98.0, 137.5)	145.5 (119.0, 197.0)	0.001 *
	LVESV (mL)	42.0 (32.0, 50.5)	52.0 (37.0, 78.5)	0.027 *
	LVEF (%)	63.0 (60.5, 68.0)	62.5 (56.5, 68.5)	0.41
	RVEDV (mL)	110.5 (86.5, 135.0)	148.5 (114.5, 188.5)	0.001 *
	RVESV (mL)	39.0 (31.0, 53.5)	53.5 (38.5, 69.5)	0.010 *
	RVEF (%)	63.5 (60.0, 68.5)	63.0 (60.0, 68.0)	0.86
	T2 Signal Ratio	2.3 (0.5)	2.1 (0.6)	0.38
	EGE	1.8 (1.1, 4.8)	2.1 (1.4, 3.0)	0.69
	LGE (% of LV mass)	2.5 (0.0, 5.0)	5.0 (0.0, 5.0)	0.17
	T2 Mapping (ms)	57.5 (54.0, 61.0)	52.0 (48.0, 55.5)	0.001 *
	Native T1 Mapping (ms)	1078.5 (1049.0, 1149.0)	1041.5 (1014.0, 1079.5)	0.003 *
	Post-Contrast T1 Mapping (ms)	353.8 (59.5)	427.6 (59.2)	<0.001 *
	ECV (%)	31.0 (29.0, 32.0)	28.0 (26.5, 30.0)	0.003 *
Pathologic Cut-Off Points for CMR Tissue Characterisation Indices				
	LGE > 0% of LV mass	21 (53%)	29 (73%)	0.065
	EGE ≥ 4	11 (28%)	7 (18%)	0.28
	Native T1 Mapping > 1050 ms	27 (68%)	18 (45%)	0.043 *
	ECV ≥ 29%	32 (80%)	17 (43%)	<0.001 *
	T2 Mapping > 55 ms	27 (68%)	10 (25%)	<0.001 *
	T2 Signal Ratio > 1.9	28 (70%)	24 (60%)	0.35
Cardiovascular Risk Factors				
	Hypertension	4 (10%)	7 (18%)	0.33
	Smoker (last 5 years)	5 (13%)	7 (18%)	0.53
	CAD/CVD Family History	3 (8%)	6 (15%)	0.29
	Hyperlipidemia	6 (15%)	6 (15%)	0.99
	Diabetes Mellitus (type 2)	4 (10%)	7 (18%)	0.330

ARD, autoimmune rheumatic disease; CMR, cardiovascular magnetic resonance; NSVT, non-sustained ventricular tachycardia; eGPA/GPA, (eosinophilic) granulomatosis with polyangiitis; CAD, coronary artery disease; ARVC, arrhythmogenic right ventricular cardiomyopathy; LV/RV, left/right ventricular; EDV/ESV, end-diastolic/-systolic volume; EF, ejection fraction; EGE/LGE, early/late gadolinium enhancement; ECV, extracellular volume fraction; CVD, cardiovascular disease.

**Table 2:** Prevalence of different patterns of myocardial fibrosis in patients with LGE with and without ARDs. The proportion of LGE patterns was significantly different between the groups (*p* < 0.0001). The vast majority of patients with ARDs and identified myocardial fibrosis on CMR had no evidence of CAD.

LGE Pattern	CMR Appearance	Patients with ARDs and LGE (*n* = 21/40)		Patients without ARDs and LGE (*n* = 29/40)	
		Proportion Identified	CAD Confirmed by XCA	Proportion Identified	CAD Confirmed by XCA
Patchy Inferolateral	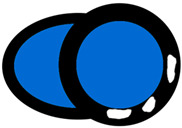	13 (61.9%)	N/A	0 (0%)	N/A
Diffuse Subendocardial	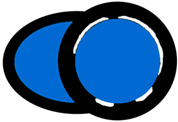	6 (28.6%)	0/6 (0%)	6 (20.7%)	0/6 (0%)
Transmural (Typical Ischaemic Pattern)	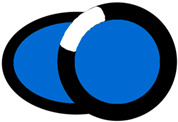	2 (9.5%)	2/2 (100%) 1 LAD 1 RCA	13 (44.8%)	13/13 (100%) 10 LAD + RCA 3 LCx
Subepicardial	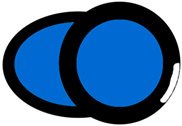	0 (0%)	N/A	12 (41.4%)	N/A

LGE, late gadolinium enhancement; CMR, cardiovascular magnetic resonance; ARD, autoimmune rheumatic disease; CAD, coronary artery disease; XCA, X-ray coronary angiography; LAD, left anterior descending coronary artery; RCA, right coronary artery; LCx left circumflex coronary artery.

**Table 3:** Logistic regression analyses for discriminating between ARD and non-ARD patients with NSVT (odds ratio > 1 means higher values more likely in patients with ARDs). All available CMR variables were individually examined and subsequently corrected first for age, hypertension, smoking, family history of CAD/CVD and hypercholesterolemia and subsequently for the same variables but with the addition of sex. * *p* ≤ 0.05.

Variable	Univariable Logistic Regression		Multivariable Logistic Regression		Multivariable Logistic Regression (+Sex)	
	OR (95% CI)	*p*-Value	OR (95% CI)	*p*-Value	OR (95% CI)	*p*-Value
LVEDV (per 5 mL)	0.90 (0.84–0.96)	0.001 *	0.90 (0.84–0.96)	0.001 *	0.93 (0.87–1.00)	0.061
LVESV (per 5 mL)	0.89 (0.80–0.98)	0.020 *	0.89 (0.80–0.99)	0.029 *	0.96 (0.86–1.08)	0.537
LVEF (per 5%)	1.06 (0.77–1.47)	0.718	1.05 (0.74–1.49)	0.766	0.86 (0.58–1.27)	0.449
RVEDV (per 5 mL)	0.91 (0.86–0.97)	0.002 *	0.90 (0.85–0.96)	0.001 *	0.95 (0.88–1.02)	0.185
RVESV (per 5 mL)	0.86 (0.77–0.97)	0.013 *	0.86 (0.76–0.97)	0.014 *	1.00 (0.85–1.17)	0.995
RVEF (per 5%)	0.89 (0.63–1.23)	0.472	0.84 (0.59–1.20)	0.342	0.59 (0.37–0.94)	0.026 *
Nat. T1 Map. (per 10 ms)	1.12 (1.04–1.21)	0.003 *	1.13 (1.05–1.23)	0.002 *	1.09 (1.01–1.18)	0.033 *
PC. T1 Map. (per 10 ms)	0.80 (0.72–0.89)	<0.001 *	0.79 (0.70–0.88)	<0.001 *	0.81 (0.72–0.91)	0.001 *
ECV (per 1%)	1.15 (1.02–1.31)	0.023 *	1.18 (1.04–1.34)	0.013 *	1.09 (0.94–1.25)	0.269
ECV > 29%	5.41 (2.00–14.66)	0.001 *	7.53 (2.51–22.6)	<0.001 *	4.90 (1.53–15.6)	0.007 *
T2 Signal Ratio (per 0.2 units)	1.07 (0.91–1.26)	0.378	1.10 (0.93–1.30)	0.284	1.04 (0.85–1.26)	0.706
T2 Mapping (per 1 ms)	1.10 (1.02–1.18)	0.009 *	1.12 (1.03–1.22)	0.005 *	1.07 (0.97–1.17)	0.165
EGE (per 1 unit)	1.10 (0.94–1.28)	0.221	1.10 (0.94–1.29)	0.247	1.13 (0.94–1.35)	0.202
LGE (per 1% of LV mass)	0.96 (0.85–1.09)	0.519	0.96 (0.85–1.09)	0.541	0.96 (0.83–1.10)	0.516

ARD, autoimmune rheumatic disease; NSVT, non-sustained ventricular tachycardia; LV/RV, left/right ventricular; EDV/ESV, end-diastolic/-systolic volume; EF, ejection fraction; Nat. T1 Map., native T1 mapping; PC. T1 Map., post-contrast T1 mapping; ECV, extracellular volume fraction; EGE/LGE, early/late gadolinium enhancement.

## Data Availability

The data underlying this article cannot be shared publicly due to reasons pertaining to the privacy of individuals that participated in the study. The data will be shared on reasonable request to the corresponding author.

## References

[1] Mavrogeni S.I., Markousis-Mavrogenis G., Aggeli C., Tousoulis D., Kitas G.D., Kolovou G., Iliodromitis E.K., Sfikakis P.P. (2019). Arrhythmogenic inflammatory cardiomyopathy in autoimmune rheumatic diseases: A challenge for cardio-rheumatology. Diagnostics.

[2] Srinivasan N.T., Schilling R.J. (2018). Sudden Cardiac Death and Arrhythmias. Arrhythmia Electrophysiol. Rev..

[3] Myerburg R.J., Reddy V., Castellanos A. (2009). Indications for Implantable Cardioverter-Defibrillators Based on Evidence and Judgment. J. Am. Coll. Cardiol..

[4] Israel C.W. (2014). Mechanisms of sudden cardiac death. Indian Heart J..

[5] Seferović P.M., Ristić A.D., Maksimović R., Simeunović D.S., Ristić G.G., Radovanović G., Seferović D., Maisch B., Matucci-Cerinic M. (2006). Cardiac arrhythmias and conduction disturbances in autoimmune rheumatic diseases. Rheumatology.

[6] Rosenstein E.D., Zucker M.J., Kramer N. (2000). Giant cell myocarditis: Most fatal of autoimmune diseases. Semin. Arthritis Rheum..

[7] Lazzerini P.E., Bertolozzi I., Acampa M., Fulceri R., Laghi-Pasini F., Capecchi P.L. (2018). Torsades de Pointes in patients with polymyalgia rheumatica. Curr. Pharm. Des..

[8] Lazzerini P.E., Capecchi P.L., Acampa M., Galeazzi M., Laghi-Pasini F. (2014). Arrhythmic risk in rheumatoid arthritis: The driving role of systemic inflammation. Autoimmun. Rev..

[9] Mavrogeni S., Gargani L., Pepe A., Monti L., Markousis-Mavrogenis G., De Santis M., De Marchi D., Koutsogeorgopoulou L., Karabela G., Stavropoulos E. (2019). Cardiac magnetic resonance predicts ventricular arrhythmias in scleroderma: The Scleroderma Arrhythmia Clinical Utility Study (SAnCtUS). Rheumatology.

[10] Mavrogeni S.I., Sfikakis P.P., Markousis-Mavrogenis G., Bournia V.K., Poulos G., Koutsogeorgopoulou L., Karabela G., Stavropoulos E., Katsifis G., Boki K. (2019). Cardiovascular magnetic resonance imaging pattern in patients with autoimmune rheumatic diseases and ventricular tachycardia with preserved ejection fraction. Int. J. Cardiol..

[11] Mavrogeni S.I., Kitas G.D., Dimitroulas T., Sfikakis P.P., Seo P., Gabriel S., Patel A.R., Gargani L., Bombardieri S., Matucci-Cerinic M. (2016). Cardiovascular magnetic resonance in rheumatology: Current status and recommendations for use. Int. J. Cardiol..

[12] Mavrogeni S., Sfikakis P., Dimitroulas T., Kolovou G., Kitas G.D. (2014). Edema and fibrosis imaging by cardiovascular magnetic resonance: How can the experience of Cardiology be best utilized in rheumatological practice?. Semin. Arthritis Rheum..

[13] Mavrogeni S., Markousis-Mavrogenis G., Koutsogeorgopoulou L., Dimitroulas T., Bratis K., Kitas G.D., Sfikakis P., Tektonidou M., Karabela G., Stavropoulos E. (2017). Cardiovascular magnetic resonance imaging pattern at the time of diagnosis of treatment naïve patients with connective tissue diseases. Int. J. Cardiol..

[14] Friedrich M.G., Sechtem U., Schulz-Menger J., Holmvang G., Alakija P., Cooper L.T., White J.A., Abdel-Aty H., Gutberlet M., Prasad S. (2009). Cardiovascular Magnetic Resonance in Myocarditis: A JACC White Paper. J. Am. Coll. Cardiol..

[15] Mavrogeni S., Anastasakis A., Sfendouraki E., Gialafos E., Aggeli C., Stefanadis C., Kolovou G. (2013). Ventricular tachycardia in patients with family history of sudden cardiac death, normal coronaries and normal ventricular function. Can cardiac magnetic resonance add to diagnosis?. Int. J. Cardiol..

[16] Robinson A.A., Chow K., Salerno M. (2019). Myocardial T1 and ECV Measurement: Underlying Concepts and Technical Considerations. JACC Cardiovasc. Imaging.

[17] Mavrogeni S., Markousis-Mavrogenis G., Kolovou G. (2014). Clinical use of cardiac magnetic resonance in systemic heart disease. Eur. Cardiol. Rev..

[18] Freitas P., Ferreira A.M., Arteaga-Fernández E., De Oliveira Antunes M., Mesquita J., Abecasis J., Marques H., Saraiva C., Matos D.N., Rodrigues R. (2019). The amount of late gadolinium enhancement outperforms current guideline-recommended criteria in the identification of patients with hypertrophic cardiomyopathy at risk of sudden cardiac death. J. Cardiovasc. Magn. Reson..

[19] Soto-Iglesias D., Penela D., Jáuregui B., Acosta J., Fernández-Armenta J., Linhart M., Zucchelli G., Syrovnev V., Zaraket F., Terés C. (2020). Cardiac Magnetic Resonance-Guided Ventricular Tachycardia Substrate Ablation. JACC Clin. Electrophysiol..

[20] Markousis-Mavrogenis G., Bournia V.K., Panopoulos S., Koutsogeorgopoulou L., Kanoupakis G., Apostolou D., Katsifis G., Polychroniadis M., Dimitroulas T., Kolovou G. (2019). Cardiovascular magnetic resonance identifies high-risk systemic sclerosis patients with normal echocardiograms and provides incremental prognostic value. Diagnostics.

[21] Nelson T., Garg P., Clayton R.H., Lee J. (2019). The role of cardiac MRI in the management of ventricular arrhythmias in ischaemic and non-ischaemic dilated cardiomyopathy. Arrhythmia Electrophysiol. Rev..

[22] Mavrogeni S.I., Sfikakis P.P., Koutsogeorgopoulou L., Markousis-Mavrogenis G., Dimitroulas T., Kolovou G., Kitas G.D. (2017). Cardiac Tissue Characterization and Imaging in Autoimmune Rheumatic Diseases. JACC Cardiovasc. Imaging.

[23] Agca R., Heslinga S.C., Rollefstad S., Heslinga M., McInnes I.B., Peters M.J.L., Kvien T.K., Dougados M., Radner H., Atzeni F. (2016). EULAR recommendations for cardiovascular disease risk management in patients with rheumatoid arthritis and other forms of inflammatory joint disorders: 2015/2016 update. Ann. Rheum. Dis..

[24] Ntusi N.A.B., Francis J.M., Sever E., Liu A., Piechnik S.K., Ferreira V.M., Matthews P.M., Robson M.D., Wordsworth P.B., Neubauer S. (2018). Anti-TNF modulation reduces myocardial inflammation and improves cardiovascular function in systemic rheumatic diseases. Int. J. Cardiol..

[25] Ntusi N.A.B., Francis J.M., Gumedze F., Karvounis H., Matthews P.M., Wordsworth P.B., Neubauer S., Karamitsos T.D. (2019). Cardiovascular magnetic resonance characterization of myocardial and vascular function in rheumatoid arthritis patients. Hell. J. Cardiol..

[26] Hinojar R., Foote L., Sangle S., Marber M., Mayr M., Carr-White G., D’Cruz D., Nagel E., Puntmann V.O. (2016). Native T1 and T2 mapping by CMR in lupus myocarditis: Disease recognition and response to treatment. Int. J. Cardiol..

[27] Aggarwal D., Singla S. (2017). Prevalence of autonomic neuropathy in patients of rheumatoid arthritis and its correlation with disease severity. J. Clin. Diagnostic Res..

[28] Josephson M.E. (2008). Clinical Cardiac Electrophysiology: Techniques and Interpretations.

[29] De Bakker J.M.T., Van Capelle F.J.L., Janse M.J., Tasseron S., Vermeulen J.T., De Jonge N., Lahpor J.R. (1993). Slow conduction in the infarcted human heart: “Zigzag” course of activation. Circulation.

[30] Estner H.L., Zviman M.M., Herzka D., Miller F., Castro V., Nazarian S., Ashikaga H., Dori Y., Berger R.D., Calkins H. (2011). The critical isthmus sites of ischemic ventricular tachycardia are in zones of tissue heterogeneity, visualized by magnetic resonance imaging. Hear. Rhythm.

[31] Nguyen T.P., Qu Z., Weiss J.N. (2014). Cardiac fibrosis and arrhythmogenesis: The road to repair is paved with perils. J. Mol. Cell. Cardiol..

[32] Dinov B., Fiedler L., Schönbauer R., Bollmann A., Rolf S., Piorkowski C., Hindricks G., Arya A. (2014). Outcomes in catheter ablation of ventricular tachycardia in dilated nonischemic cardiomyopathy compared with ischemic cardiomyopathy: Results from the Prospective Heart Centre of Leipzig VT (HELP-VT) Study. Circulation.

[33] Chen Z., Sohal M., Voigt T., Sammut E., Tobon-Gomez C., Child N., Jackson T., Shetty A., Bostock J., Cooklin M. (2015). Myocardial tissue characterization by cardiac magnetic resonance imaging using T1 mapping predicts ventricular arrhythmia in ischemic and non-ischemic cardiomyopathy patients with implantable cardioverter-defibrillators. Hear. Rhythm.

[34] Wong T.C., Piehler K., Meier C.G., Testa S.M., Klock A.M., Aneizi A.A., Shakesprere J., Kellman P., Shroff S.G., Schwartzman D.S. (2012). Association between extracellular matrix expansion quantified by cardiovascular magnetic resonance and short-term mortality. Circulation.

[35] Greulich S., Kitterer D., Latus J., Aguor E., Steubing H., Kaesemann P., Patrascu A., Greiser A., Groeninger S., Mayr A. (2016). Comprehensive Cardiovascular Magnetic Resonance Assessment in Patients with Sarcoidosis and Preserved Left Ventricular Ejection Fraction. Circ. Cardiovasc. Imaging.

[36] Gin P.L., Wang W.C., Yang S.H., Hsiao S.H., Tseng J.C. (2006). Right Heart Function in Systemic Lupus Erythematosus: Insights from Myocardial Doppler Tissue Imaging. J. Am. Soc. Echocardiogr..

[37] Bezante G.P., Rollando D., Sessarego M., Panico N., Setti M., Filaci G., Molinari G., Balbi M., Cutolo M., Barsotti A. (2007). Cardiac magnetic resonance imaging detects subclinical right ventricular impairment in systemic sclerosis. J. Rheumatol..

[38] Velangi P.S., Chen K.H.A., Kazmirczak F., Okasha O., von Wald L., Roukoz H., Farzaneh-Far A., Markowitz J., Nijjar P.S., Bhargava M. (2020). Right Ventricular Abnormalities on Cardiovascular Magnetic Resonance Imaging in Patients With Sarcoidosis. JACC Cardiovasc. Imaging.

